# Effects of 3 Different Commercial Vaccines Formulations against BVDV and BHV-1 on the Inflammatory Response of Holstein Heifers

**DOI:** 10.3390/vetsci6030069

**Published:** 2019-08-26

**Authors:** Camila Costa Baccili, Camila Cecilia Martin, Nathália Decaris, Karina Medici Madureira, Christopher Chase, Viviani Gomes

**Affiliations:** 1 Department of Internal Medicine, College of Veterinary Medicine and Animal Science, University of Sao Paulo, 87, Prof. Dr. Orlando Marques de Paiva Avenue, Cidade Universitária, Butantã, Sao Paulo 05508-270, Brazil; 2 Department of Anatomy, Pathology and Clinical Medicine, Federal University of Bahia, 500, Adhemar de Barros Avenue, Ondina, Salvador 40170-110, Brazil; 3 Department of Veterinary and Biomedical Sciences, South Dakota State University, Brookings, SD 57007, USA

**Keywords:** vaccine formulation, adverse reactions, adjuvants, inflammation, antibodies

## Abstract

After vaccination, vaccine components must activate the immune response, but the ideal vaccine should not result in undesirable effects in cattle. The aim of this study was to evaluate the inflammatory and humoral responses and adverse reactions induced by three adjuvanted commercial vaccines against bovine viral diarrhea virus (BVDV) and bovine herpesvirus 1 (BHV-1). Holstein heifers (*n* = 35) were divided into four groups by adjuvant compounds: Vaccine A (Alum; *n* = 9), Vaccine B (Oil-in-water; *n* = 10), Vaccine C (Amphigen/Quil A cholesterol and dimethyl-dioctadecyl ammonium (DDA) bromide (QAD; *n* = 10), and Control (*n* = 6). Heifers were assessed at 0 h, 6, 24, 48, 72 and 168 h post-vaccination; serology was evaluated at first dose (D0), booster (D21) and D42. Heifers vaccinated with Vaccine B (*p* = 0.0001) and C (*p* = 0.0001) had a more intense local reaction, while there was a higher rectal temperature detected in heifers vaccinated with Vaccine C (*p* = 0.020). There was greater systemic reaction observed for heifers vaccinated with Vaccines B and C at 48 h (*p* = 0.002) after a second dose. Clinical pathology parameters [white blood count (WBC) (*p* = 0.001), neutrophils (*p* = 0.0001) and haptoglobin concentrations (*p* = 0.0001)] were higher in animals vaccinated with Vaccine C. Neutralizing Abs against BVDV type 1 strains, NADL and Singer, were detected in animals vaccinated with Vaccines A or C at D42, while BVDV-2 antibodies were detected only in animals vaccinated with Vaccine C. A BHV-1 antibody was detected in all three vaccine groups (Vaccines A, B or C) at day 42 (21 days post booster vaccination). The findings of this research were based on three different commercial laboratory formulations and also according to the conditions which the study was conducted. In this context, vaccine containing mineral oil or Amphigen/QAD presented greater local reactivity and induced a significant systemic inflammatory response. Vaccinated heifers with Alum and Amphigen/QAD commercial vaccines enhanced humoral immune response against BVDV and BHV-1.

## 1. Introduction

Bovine viral diarrhea virus (BVDV) and bovine herpesvirus 1 (BHV-1) cause significant economic losses for cattle producers on a global scale, because of reproductive and/or respiratory disease that can result in morbidity, mortality and decreased milk production. These viruses can serve as a barrier to the export of live cattle to regions or countries within Europe that have already eradicated these viral diseases [1,2,3].

Herd vaccination during pre-breeding or the breeding period is used as a prevention program to reduce reproductive loss associated with BVDV and BHV-1 infections. These two viruses affect reproduction by decreasing conception rates, causing early embryonic death and/or abortion [4]. In addition, BVDV infections in the first trimester can result in persistently infected (PI) animals. These PI animals often have a shortened life span but more importantly these animals are a constant threat to the health of herd as they are a continual source of infection to the herd. The prevalence of BVDV PI animals are considered low (≤0.8%) in Europe, North America and Australia. The BVDV PI and antibody-positive prevalence in Europe has decreased over time, while BVDV prevalence has increased in North America. Higher PI prevalence occur in countries that had failed to implement any BVDV control and/or eradication programs (including vaccination) [5].

Reproductive vaccination protocols present a strategy to limit reproductive losses among dairy and beef herds. However, the difference between the action of the vaccine and effective immunization of the herd must be taken into consideration. For an animal to be effectively vaccinated, it must develop a solid T cell and B cell response. For the herd to develop herd immunity against these two viruses, about 70–80% of the herd needs to develop an immune response to result in effective herd immunity [6].

The cascade of events occurring after vaccination begins with the activation of the innate immune system by dendritic cells (DCs) interacting with specialized pattern recognition receptors (PRRs), including toll-like receptors (TLRs) [7,8]. The TLR-type receptors for BHV-1 and BVDV are TLR3 and TLR7 [9,10]. Initially macrophages and/or DCs secrete proinflammatory cytokines, primarily interleukin-1 beta (IL-1β), interleukin-6 (IL-6), and tumor necrosis factor alpha (TNF-α) [11]. These cytokines then recruit and/or activate antigen presenting cells (APCs), including macrophages, monocytes, and more DCs, at the site of inflammation, then, enhancing antigen presentation capacity, and migration to lymphoid tissues where the DCs interact with T cells and B cells to initiate and develop the adaptive immune response [12,13].

The presentation of antigen by APCs to the naive TCD4^+^ lymphocytes (first interaction) in the lymph nodes, followed by signaling, results in the activation of the cell-mediated immune response. These lymphocytes subsequently differentiate into T helper 1 (Th1) or T helper 2 (Th2) effector T cells. The TCD8^+^ lymphocytes and B-lymphocytes migrate from the thymus and bone marrow, respectively, to regional lymph nodes, where secondary immune interaction occurs between CD4^+^ type-Th1, CD8^+^-cytotoxic T cells (CTL) or CD4^+^ type-Th2, and B lymphocytes. After these interactions, TCD8^+^ and B cells become effector or memory cells [12,14,15].

The key to an effective vaccine response is the activation of many DCs for amplification of the cellular interaction between APCs and helper T lymphocytes in lymph nodes. One of the ways to activate DCs is with the additional of adjuvants to the vaccine. This is essential for vaccines containing inactivated antigens. Different adjuvants have different types of activation and levels of local inflammation, stimulating the production of high levels of proinflammatory cytokines IL-1, IL-6, and TNF-α. These cytokines result in a local effect, resulting in the release of other cytokines that recruit and activate additional cells that are important for antigen presentation and the activation of the acquired immune response [11]. The release of IL-1β, IL-6 and TNF-α at an inflammatory site is increased due to the paracrine effect; the cytokines not only bind to the TLR receptors of the APCs that produced it, but also to neighboring cell receptors. This results in a systemic release of cytokines in the circulation. In this way the inflammation state is generated, because the cytokines reach the hypothalamus and other central nervous system (CNS) centers, stimulating the production of prostaglandin-E2, which induces hyperthermia (pyrexia), listlessness (attitude changes) and reduced appetite [7,8,11,16].

Since inflammation is a key part of the activation of innate immune system by vaccination and/or antigenic stimulation, the ability to modulate (control) this response is essential. Here the “bad” effects of the inflammation (fever, listlessness and inappetence) occur, but the immune system is regulated by anti-inflammatory response to minimize the “negative” responses to the vaccine that can lead to impaired immunity, decreased dry matter intake, reduced weight gain, metabolic disorders (acidosis and ketosis), reduction in milk production and decreased overall animal welfare [17]. 

Additionally, there are concerns about the potential risk for adverse events following vaccination. Vaccines may trigger anaphylactic, anaphylactoid or endotoxic shock reactions in animals following vaccinations, which can occur within minutes or hours after administering the vaccine. This results in systemic reactions characterized by lethargy, hyperthermia, dyspnea, tremors, pulmonary edema, abortion, and even death [18]. Anaphylaxis is an immune-mediated allergic reaction caused by the release of inflammatory mediators triggered by binding of an immunogen to immunoglobulin E (IgE)-bound to mast cells and basophils. This reaction requires prior exposure to an agent or chemical. Anaphylactoid reactions are clinically similar to anaphylaxis, but do not have an IgE component and occur without prior sensitization [19].

Activation of the innate immune system by adjuvants result in a greater immune response. However, over stimulation or stimulation that elicits an inappropriate response results in adverse reactions. The ideal adjuvant would promote a better immune response specific to the vaccine antigen reflected by the development of neutralizing antibodies and amplify the cell-mediated immune response with no to mild injection site reactions [20]. Although local and systemic inflammatory responses are generated by adjuvants present in vaccines, there is limited evidence comparing different vaccine and adjuvant formulations [21]. With the aim to elucidate the inflammatory response induced by the most frequently used immunogens, we hypothesized that different formulations of the commercial vaccines with three based of adjuvants would induce different degrees of inflammation. The objective of this study was to evaluate the intensity of adverse reactions, inflammatory and humoral response induced by three different formulation adjuvanted vaccines against BVDV and BHV-1.

## 2. Materials and Methods

### 2.1. Animals

This research was approved by University of Sao Paulo Animal Care and Use Committee (Approval number: 6229201216). Fifteen (15) to 24 months old Holstein heifers from the Sao Paulo Agency of Agribusiness Technology (APTA)-Dairy Cattle herd, located in the city of Nova Odessa—Sao Paulo (22°75′S of latitude and 47°27′W of longitude) were used. This farm was selected because the animals had not been previously vaccinated for BVDV and BHV-1. The only preventative health protocol used on the farm was the foot-and-mouth disease and brucellosis vaccination.

The study was initiated with the identification of naïve BVDV and BHV-1 heifers. Serum and whole blood samples were collected from 38 heifers in the herd. The serum was used to detect BVDV types 1a (BVDV strain NADL and Singer) and BHV-1 (Los Angeles strain) antibodies by serum virus neutralization (SVN). RNA was extracted from the whole blood samples and BVDV reverse transcriptase polymerase chain reaction (RT-PCR) was performed [22]. BVDV RT-PCR was repeated 21 days later to distinguish between BVDV transient or persistent infection. During the pre-vaccination period, 3 heifers were sold, leaving 35 heifers that tested BVDV negative on SVN and RT-PCR.

The heifers were fed a standard of care diets with water and ad libitum mineral salt. The reproductive status of the selected heifers was obtained through herd records. The breeding synchronization protocol had not been used in 24 of 35 heifers; 6 of the heifers were approximately 90 days pregnant; and 5 had been bred one month before the start of the study.

### 2.2. Vaccination and Management

The heifers (*n* = 35) were randomized and divided into four experimental groups: Vaccine A (Alum; *n* = 9), Vaccine B (Oil-in-water; *n* = 10), Vaccine C (Amphigen and Quil A cholesterol and dimethyl-dioctadecyl ammonium (DDA) bromide (QAD) adjuvant; *n* = 10), and Control (*n* = 6) (Table 1). The vaccines were administered subcutaneously in the right side of the neck using a 1.20 × 40 mm single sample needle (Precision Glide^®^, BD Diagnosis, Franklin Lakes, NJ, USA) into syringe of 5 mL (Platispack^®^, BD Diagnosis, Franklin Lakes, NJ, USA).The heifers received two doses of vaccines (5 mL), at a 21-day interval and unvaccinated group (control) received saline injection (5 mL) at the same 21day interval. The batch vaccines used for first and second dose: Vaccine A (Lot:001/15), Vaccine B (Lot:003/15), Vaccine C (Lot:002/15).

**Figure 1:**
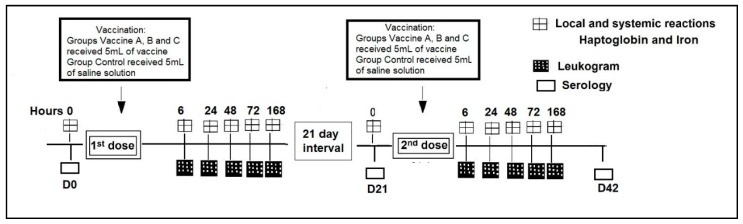
Timeline of experimental design.

The heifers were evaluated for adverse effects, serum haptoglobin (Hp) and iron concentrations at (0 h) and after vaccination at 6, 24, 48, 72, and 168 h. A complete leukogram, including: Total leukocytes, lymphocytes, monocytes, basophils, neutrophils, eosinophils and platelets was performed after vaccination at 6, 24, 48, 72, and 168 h (Figure 1). Specific neutralizing antibodies against BVDV and BHV-1 were measured at the day (D) of vaccination day 0 (D0), at booster day 21 (D21) and 42 days post vaccination (D42). The interval between the first and second dose of vaccine was 21 days.

### 2.3. Local and Systemic Reaction

Injection site reactions were evaluated by inspection and palpation to detect the cardinal signs of inflammation: Zero (0)—absence of signs, and (1) presence of pain, heat, and redness. Additionally, skin measurements were performed in all experimental groups at the vaccination site on the injection triangle for subcutaneous application. The height and width of the injection site reactions was measured using a caliper and was expressed in cm, then were transformed into area (height × width) and expressed in square millimeter (mm^2^).

The systemic reaction of heifers was assessed using the University of Wisconsin Calf Heath Scoring Criteria [23]. Fecal scores were assigned on a scale of 0–3 [23]. Respiratory effects were scored using a combination of the following parameters: rectal temperature, cough, nasal and ocular secretion with a score of 0–3 for each parameter based on severity of each [23]. The heifers were assessed as having a significant systemic reaction when the sum of these scores was >5.0. The per acute anaphylactoid vaccination reactions were assessed: 0-absence of signs, 1-Anaphylactoid/anaphylactic reaction. The same person performed and assessed the systemic and local reactions.

### 2.4. Blood Samples

Blood samples were collected via coccygeal venipuncture using a 25gauge × 0.8-cm single sample needle (Vacutainer^®^, BD Diagnosis, Franklin Lakes, NJ, USA) into vacuum tubes (Vacutainer^®^, BD Diagnosis, Franklin Lakes, NJ, USA) with and without anticoagulant to obtain blood and serum, respectively. Blood samples were collected on study days at 0, 6, 24, 48, 72 and 168 h in a 2 mL polypropylene tube for assessing leukocyte and platelet counts (BD Vacutainer K2 Ethylenediaminetetraacetic acid - EDTA, 3.6 mg REF367841^®^; BD Diagnosis, Franklin Lakes, NJ, USA) and into two individual 10 mL glass tubes (BD Vacutainer Serum^®^; BD Diagnosis, Franklin Lakes, NJ, USA) for testing serum iron, Hp and antibody concentration.

### 2.5. White Blood Count (WBC)

The EDTA blood samples were transported in a cooler at 4 °C and leukocyte and platelets counts analyzed in the Clinical Medicine Department, University of Sao Paulo (USP), College of Veterinary Medicine and Animal Science in Sao Paulo-SP. The total leukocyte, platelet and differential leukocyte counts for each sample were determined by the use of an automatic cell counter (ABC Vet, Horiba ABX^®^, Montpellier, France).

### 2.6. Iron Concentration

The serum iron concentration was determined using a commercial kit (Unsaturated Iron Binding Capacity - UIBC, (CTLF), SI250, Randox Laboratories Ltd., Co. Antrium, UK) according to manufacturer instructions. The concentration values were expressed in micromolar per liter (µM/L).

### 2.7. Haptoglobin

Haptoglobin (Hp) levels were measured using the methodology [24], adapted by [25]. A standard curve was developed with standard Hp solutions diluted from 0.56 to 0.01 g/L. Fifty μL of Hp standard or serum sample was added to 50 μL of 0.9% saline solution in each well. Next, 50 μL of sheep methemoglobin solution (30 mg/dL) was added and plates were incubated for 10 min at 20 °C. A blank (50 μL of 0.9% saline) was run with each serum sample. Following incubation, a guaiacol reagent (150 μL, pH 4.0) and 50 μL of H_2_O_2_ solution (0.02 mol/L) were added. After 10 min, absorbance at 490 nm was measured using a microplate reader. All samples were run in duplicate and the mean of each duplicate was used to calculate the final concentration based on the standard curve.

### 2.8. Serum Virus Neutralization (SVN)

Neutralizing antibodies against BVDV-1 (NADL) and BHV-1 (Los Angeles) were performed according to the recommendations of the “Manual of Diagnostic Tests and Vaccines of Terrestrial Animals” with modifications [26], at the Laboratory of Bovine Viruses of the Biological Institute of Sao Paulo. Dilution of serum in log_2_ were done following a 1:10 (BVDV-1 NADL strain) or 1:2 (BHV-1-Los Angeles strain) dilutions using the cell culture medium MEM (minimum essential medium) containing 1% of Pen-Strep antibiotics and 5% BVDV Ab-free fetal bovine serum as the diluent. An amount of 50μL of serum in duplicates were added to wells of the plates, after which 50 μL of the respective virus solution containing TCID_50_/100 μL (50% tissue culture infective doses) was added. Plates were incubated for 18–24 h for BHV-1 and one hour for BVDV in a 37 °C oven with 5% CO_2_. After incubation the MDBK (Madin-Darby bovine kidney) cell suspension was added to each well of the plates. The serum neutralization tests for BVDV-1 Singer strain and BVDV-2 VS253 strain were done at the Department of Virology at University of Santa Maria using 100 to 200 infecting doses of each virus used. After this procedure the plate was incubated at 37 °C with 5% CO_2_ for 4–5 days. Viral infectivity was indicated by the cytopathic effect (CPE) visible in the cell monolayer under inverted microscope. Antibody titer was expressed as the highest dilution of serum that completely inhibited the CPE in both wells of each dilution. Virus titer was determined by retitration [27].

### 2.9. Statistical Analysis

Analysis of data was carried out using the Statistical Analysis System (SAS^®^ version 9.4, SAS Institute, Cary, NC, USA). All variables were evaluated for Gaussian distribution by function guided data analysis. Some data presented no normal distribution and were subjected to a logarithmic transformation by log_10_, square root or inverse to obtain a normal distribution of the variables.

Variables were tested for fixed effects of treatments (P_Tr_; Vaccines A, B, C and Control) and days (P_Day_; 0, 6, 24, 48, 72 and 168 h), as well as the interaction of treatment and day (P_TrxDay_; effects by a MIXED procedure (PROC-mixed, SAS), with least significant difference (LSD) post hoc test. The models were tested according to covariance structures, using Akaike information criterion (AIC). Differences were considered significant when *p* ≤ 0.05.

Qualitative data (scores) were presented in frequency values, the comparison between groups evaluated with Chi-square test and the times by Cochran’s Q test. Multiple analyses between the time intervals were carried out with McNemar’s post hoc test with Bonferroni correction (*p* = 0.003) was considered to indicate statistical significance (*).

## 3. Results

### 3.1. Local Vaccine Reactions

Heifers manifested localized pain and heat at the injection site in each of the experimental groups after vaccination (first dose) and booster (second dose) (Table A1). From the cardinal inflammatory signs, it was possible to observe differences between groups at 6 h (*p* = 0.012), 24 h (*p* = 0.044), 72 h (*p* = 0.013) and 168 h (*p* = 0.041) after the first dose; a higher frequency of local inflammation was detected in heifers vaccinated with Vaccine B. After the booster, animals vaccinated with Vaccine C had greater local inflammation at 6 h (*p* = 0.001), 24 h (*p* = 0.015), 48 h (*p* = 0.002) and 168 h (*p* = 0.042).

Time analysis by Cochran’s test in each group showed differences only in vaccinated heifers. After first and second doses, Vaccine A and C group were more reactive to painful palpation at 6 h with a gradual decrease up to 168 h post vaccination (Table A1). In contrast, heifers receiving Vaccine B remained reactive between 6 h and 168 h (*p* ≤ 0.050). Multiple analysis between time intervals with post hoc test (McNemar) with Bonferroni correction did not reveal differences between intervals for any group (*p* ≥ 0.016) (Table A2).

**Figure 2:**
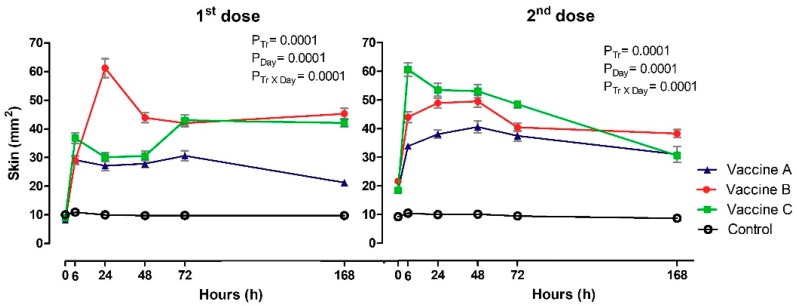
The figure represents the mean values and standard deviations of local vaccine reaction with skin thickness at the injection site in animals vaccinated with three different formulation adjuvanted vaccines against BVDV and BHV-1. Overall statistical significance (*p* ≤ 0.05) is indicated in the plot as follows: Effects of treatment (P_tr_), day (P_Day_) and interactions (P_tr_ × P_Day_).

The heat variable showed differences between the groups at 6 h (*p* = 0.019), 24 h (*p* = 0.001), 48 h (*p* = 0.035) and 72 h (*p* = 0.012) after the first dose, with a higher heat-positive frequency of heifers in Vaccines B and C compared to Vaccine A and Control groups (Table A1). It was not possible to detect differences between treatments after booster. Cochran’s test showed variations in Vaccines A, B and C groups after first and second doses of vaccines, with higher localized heat reactions after the first dose at 24 h for Vaccine C, 48 h for Vaccine B and later at 72 h in the Vaccine B group. Following the second dose, higher localized temperature was observed in most heifers vaccinated with Vaccine C (6 h), Vaccine A (24 h) and Vaccine B (48 h). The McNemar post hoc test revealed no differences for any of the experimental groups (*p* ≥ 0.008). No animals exhibited rubor (redness) (data not shown). The skin thickness increased following vaccination in the vaccinated heifers (Figure 2). Differences were observed between groups and day and it was possible to detect interaction Tr x Day after the first and second doses of vaccines (*p* ≤ 0.05). Skin measurements allowed identification of volume increase at point of application in all heifers vaccinated in relation to control group, after application of first dose between 6 h and 168 h. Maximum increase in skin thickness was observed for Vaccine B (*p* = 0.0001) at 24 h (61 mm^2^), compared to Vaccine A (27 mm^2^) and Vaccine C (30 mm^2^) after the first dose. In contrast, for the second dose of vaccine, there was an increase in skin thickness for Vaccine C (*p* = 0.0001; 61 mm^2^) compared to Vaccine A (34 mm^2^) and Vaccine B (44 mm^2^). The skin thickness of heifers control group was significantly less (ranged from 9 to 11mm^2^) than vaccinated heifers at 6h after the first dose and then at all times after the second dose.

### 3.2. Systemic Vaccine Reactions

None of the vaccinated heifers had any anaphylactoid or anaphylactic reactions after the first or second doses of the vaccines at any time following vaccination.

**Figure 3:**
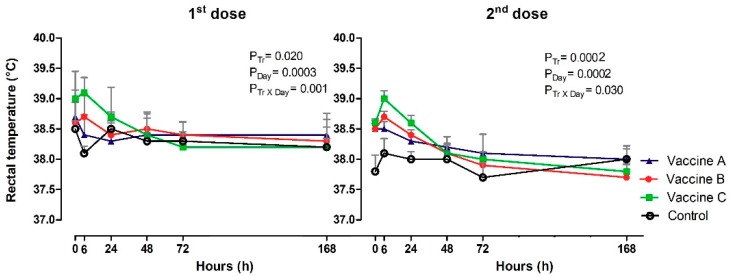
The figure represents the mean values and standard deviations of systemic vaccine by rectal temperature (°C) in animals vaccinated with three different formulation adjuvanted vaccines against BVDV and BHV-1.Overall statistical significance (*p* ≤ 0.05) is indicated in the plot as follows: Effects of treatment (P_tr_), day (P_Day_) and interactions (P_tr_ × P_Day_).

There were some increases in rectal temperatures after primary and booster vaccination (Figure 3). There was a significant difference after the firstand second doses in the effect of treatment (*p* = 0.020; *p* = 0.0002), day (*p* = 0.0003; *p* = 0.0002) and Tr x Day (*p* = 0.001; *p* = 0.030), respectively. The rectal temperature at 0 h—first dose was higher in Vaccine C (39.0 °C) compared to Vaccine B (38.6 °C) and the control (38.5°). At 6 h, heifers that received Vaccine C (39.1 °C; *p* = 0.020) was higher at 6 h and control group was lowest (38.1 °C) and differed from Vaccine A (38.4 °C), B (38.7 °C). At the booster time (0 h), Vaccine A (38.5 °C), Vaccine B (38.6 °C) and Vaccine C (38.6 °C) was similar, but the control was different (37.8 °C), then, at 6 h, Vaccine B was 38.7 °C and Vaccine C (39.0 °C). Time analysis showed a decrease in body temperature at 6 h in Vaccine A group, 24 h with Vaccine B, 48 h with Vaccine C and 24 h Control. After the booster, the rectal temperature was lowest in the control group from 24 h.

Similar frequencies of systemic signs were observed between the experimental groups (*p* ≤ 0.050) receiving Vaccines A, B C or control groups (Table A2).Cochran’s test detected difference (*p* = 0.014) in coughing, only for Vaccine B group after the application of the first dose of the vaccine, where 10% of the heifers had a demonstrated cough at 24 h, 30% at 72 h (30%) and 20% at 168 h (20%).

The production of nasal secretions was different between groups at 72h (*p* = 0.051) after application of second dose, with higher numbers in Vaccine B and C animals. Differences between time frequencies were observed in Vaccine B group, Vaccine C group and control after the second dose of vaccines.

Time analysis for ocular secretions showed differences at the first dose between 0 h and 168 h for Vaccine B (*p* = 0.002) and Vaccine C (*p* = 0.018). After the booster, it was between 48 h and 72 h for Vaccine C (*p* = 0.014).

Rubor (redness) symptoms, rectal temperature and fecal score had no differences between the groups and time intervals (*p* ≥ 0.050).

The multiple comparisons between times by McNemar post hoc test with Bonferroni correction showed no significant difference in relation to the parameters of cough (*p* ≥ 0.125), nasal secretion (*p* ≥ 0.063), ocular secretion (*p* ≥ 0.031), temperature (*p* ≥ 0.125) and fecal (*p* ≥ 0.125).

Analysis of systemic effects ≥5 revealed differences between the groups at 72 h after the booster and demonstrated more heifers with higher clinical scores in Vaccine B and Vaccine C groups (*p* = 0.024). Time analysis revealed frequency variations in Vaccine C and control groups, with peak frequency observed at 48 h after application of the booster (Table A2).

### 3.3. White Blood Count (WBC)

**Figure 4:**
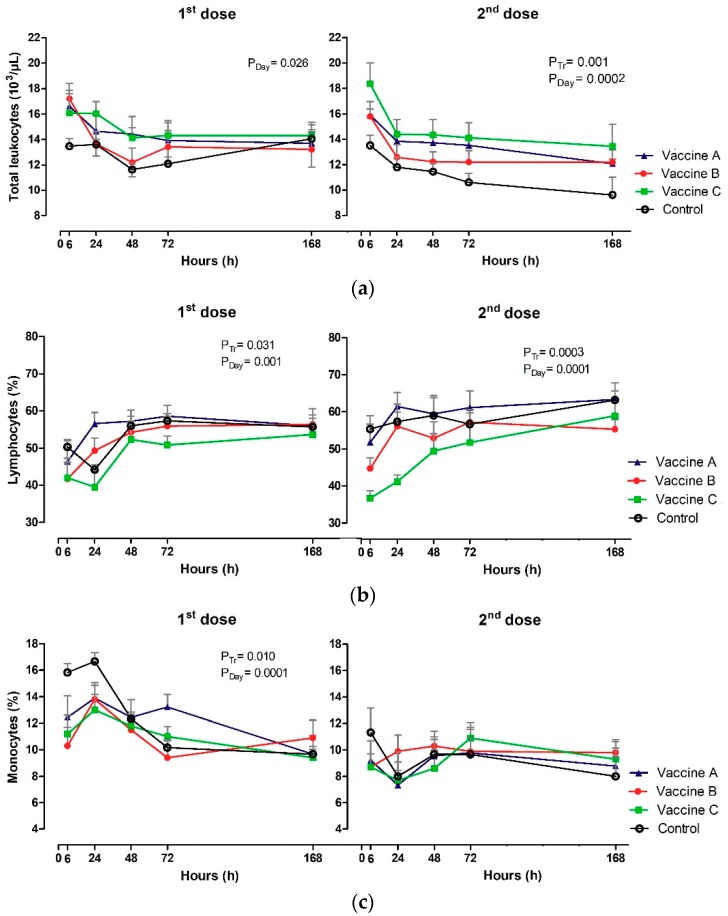

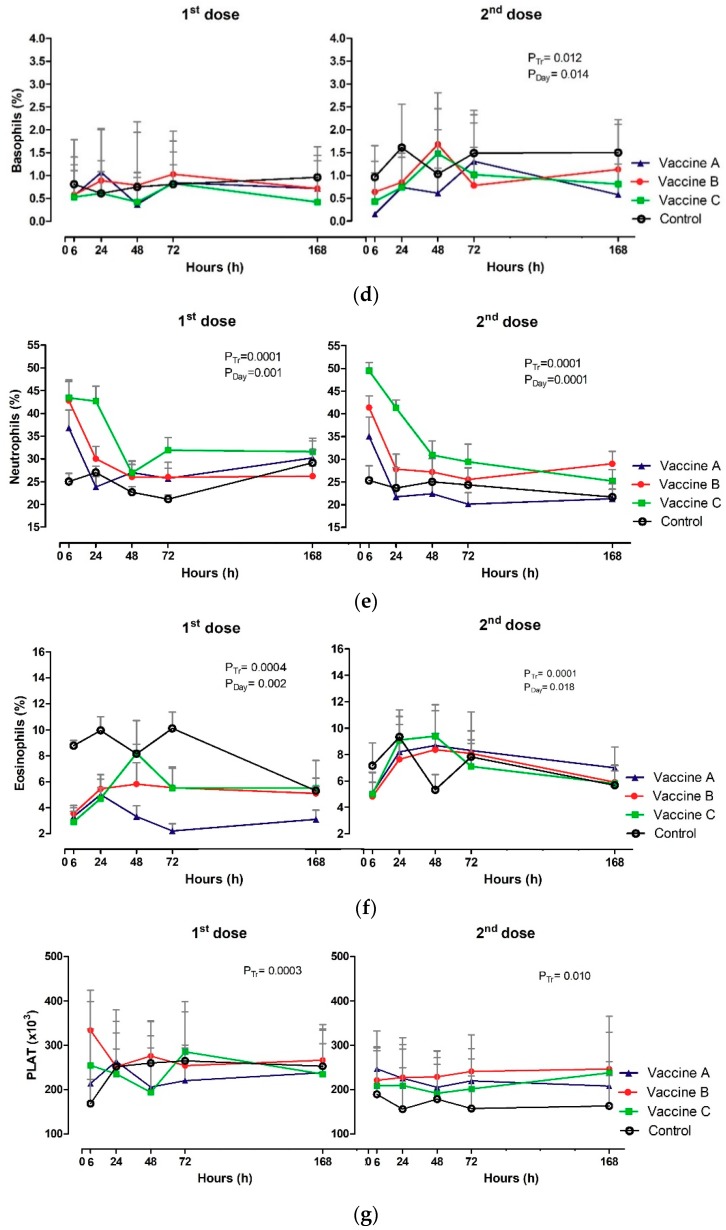
Effect of vaccination on Clinical pathology: (**a**) Total leukocytes (×10^3^/µL); (**b**) lymphocytes (%); (**c**) monocytes (%); (**d**) basophils (%); (**e**) neutrophils; (**f**) eosinophils (%), (**g**) and platelets (×10^3^/µL) in animals vaccinated with three different formulation adjuvanted vaccines against BVDV and BHV-1. Reported are the mean values and standard deviations. Overall statistical significance (*p* ≤ 0.05) is indicated in the plot as follows: effects of treatment (P_tr_), day (P_Day_) and interactions (P_tr_ × P_Day_).

The profile of leukograms and platelet counts are shown in Table A3 and Table A4; Figure 4a–g. The WBC (×10^3^/µL) had no treatment effect or Tr x day interaction after the first dose of the vaccine (*p* ≥ 0.050) (Figure 4a). On the other hand, the effect of day on the number of total leukocytes (*p* = 0.026) was detected, with a decrease in WBC in Vaccine B group between 6 h and 168 h. Regarding the second dose, only the treatment effect (*p* = 0.001) and the day (*p* = 0.0002) were observed. The maximum number of WBC was observed at 6 h in vaccinated and control groups, with a gradual decrease in subsequent moments. WBC number was higher in Vaccine C and lower in control group at 6 h and 168 h after the vaccination protocols.

Heifers vaccinated with Vaccine A had increased lymphocyte counts at 24 h after the first (*p* = 0.031), and second dose (*p* = 0.0003), and it is important to emphasize that this group had early lymphocytosis when compared to other groups. Absolute lymphocytes during first and second doses presented similar dynamics. In contrast, the proportion of lymphocytes (%) influenced treatment for the first (*p* = 0.031) and second dose including the day (*p* ≤ 0.050) (Figure 4b).

Relative values were influenced only after the first vaccination, control heifers presented higher values of monocytes at 6 h and 24 h in relation to vaccinated groups (*p* = 0.010). The control heifers, Vaccine B and Vaccine C had a gradual decrease in monocyte count up to 168 h (*p* = 0.0001) (Figure 4c). Absolute numbers of monocytes differed only in relation to the effect of the day after the first vaccination (*p* = 0.0002), higher values were observed in the Vaccine A group at 24 h (1.99 × 10^3^/µL). 

All groups had a gradual decrease in neutrophil, with greater values at 6 h after vaccination. The Vaccine C group was different (*p* = 0.001) in relation to heifers that received Vaccine A, Vaccine B or the control vaccine (Figure 4e). Absolute and relative neutrophils varied according to treatment effect and day in relation to the first vaccination, administering the booster and with time intervals (*p* ≤ 0.05).

Platelet concentrations (PLAT) were affected immediately after each vaccination-first vaccination with Vaccine B group had higher concentration at 6 h (*p* = 0.0003) and then at 72h after the Vaccine B booster (*p* = 0.010), in relation to the control group of heifers (Figure 4g).

### 3.4. Serum Iron Levels

**Figure 5:**
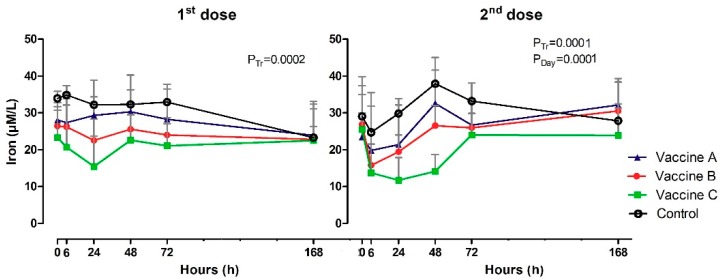
Clinical pathology by serum iron levels (µM/L) in animals vaccinated with three different formulation adjuvanted vaccines against BVDV and BHV-1. Reported are the mean values and standard deviations. Overall statistical significance (*p* ≤ 0.05) is indicated in the plot as follows: Effects of treatment (P_tr_), day (P_Day_) and interactions (P_tr_x P_Day_).

Iron concentrations (µM/L) are shown in Table A3 and Figure 5. Before vaccinations, iron serum concentrations were the same across the time intervals and groups (0h), but between 6 h and 72 h the values were lower in the vaccinated groups than in the control group (*p* = 0.0002; *p* = 0.001). The time analysis revealed small oscillations in the iron content for control group, observing the lowest value at 6 h and the highest at 48 h after booster (*p* = 0.0001). The group immunized with Vaccine C showed a significant decrease (*p* = 0.0001) in serum iron levels between 6 h and 48 h, with a slight increase in the subsequent time intervals of 72 h and 168 h.

### 3.5. Haptoglobin Inflammation

**Figure 6:**
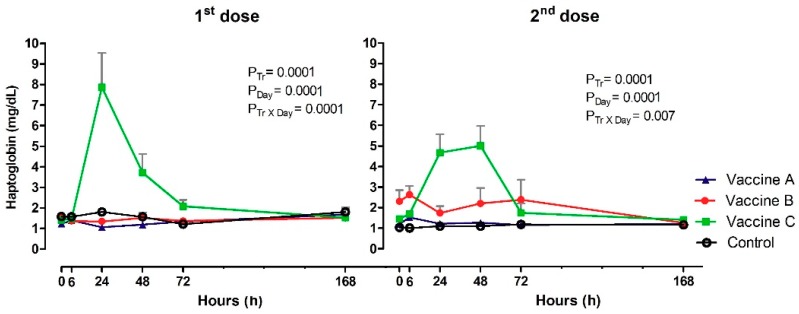
Acute phase protein production from haptoglobin concentrations (mg/dL) in animals vaccinated with three different formulation adjuvanted vaccines against BVDV and BHV-1. Reported are the mean values and standard deviations. Overall statistical significance (*p* ≤ 0.05) is indicated in the plot as follows: Effects of treatment (P_tr_), day (P_Day_) and interactions (P_tr_ × P_Day_).

Serum Hp (mg/dL) did vary by vaccination after the first and second doses in the effect of treatment (*p* = 0.0001; *p* = 0.0001), day (*p* = 0.0001; *p* = 0.0001) and Tr x Day (*p* = 0.0001; *p* = 0.007). Significant differences were observed between experimental groups after 24 h at first dose. Vaccine C group had high concentrations of Hp (7.87 mg/dL), compared to Vaccine A (1.06 mg/dL), Vaccine B (1.34 mg/dL) or the control group (1.80 mg/dL). Subsequently, there was an increase at 48 h for Vaccine C (3.71 mg/dL). At subsequent time points, no differences were observed between vaccines, although serum Hp levels in Vaccine C group were still visually higher than those in other experimental groups (Figure 6). The profile of Hp after booster was very similar to first vaccination, with higher concentrations in Vaccine C group at 24 h (4.67 mg/dL) and 48 h (5.01 mg/dL), compared with other vaccines and the control. Difference between time intervals were observed only in Vaccine B or Vaccine C groups, with maximum values at 6 h (2.63 mg/dL) and 72h (2.39 mg/dL), 24 h (4.67 mg/dL) and 48 h (5.01 mg/dL).

### 3.6. BVDV and BHV-1 Antibody Production

Differences were observed between groups, day and interaction Tr × Day after the first and second doses of vaccines (*p* ≤ 0.05) for BVDV-1 (NADL e Singer), BVDV-2 and BHV-1.

None of vaccinated groups A, B and C seroconverted to BVDV-1 NADL strain after the first dose (D21), then D42 the Abs were similar vaccine A (GMT-log_2_ = 5.1) and vaccine C (GMT-log_2_ = 5.1). Heifers receiving vaccine A seroconverted to BVDV-1 Singer strain (GMT log_2_ = 0.1) at D21. At booster day (D42) group vaccine A had higher mean titers of Abs (GMT-log_2_ = 5.8) to vaccine C (GMT-log_2_ = 3.5). Vaccine B did not respond to this strain of BVDV-1.

The BVDV-2 (VS253) SVN test revealed that only heifers receiving vaccine C produced Abs after the firstdose (GMT-log_2_ = 1.0) and the second vaccine dose (GMT-log_2_ = 6.7).

The BHV-1 Abs were detected by Vaccine A (GMT log_2_ = 2.5) and C (GMT log_2_ = 0.7) at D21. Higher response was observed in Vaccine C (GMT-log_2_ = 6.1), followed by vaccine A (GMT-log_2_ = 4.3), vaccine B (GMT-log_2_ = 2.7) at D42.

## 4. Discussion

This study evaluated the influence of commercial vaccine formulations with different types of adjuvants on local and systemic inflammation, along with adverse effects in heifers during the vaccination against BVDV and BHV-1. Vaccinated heifers showed higher local reactivity, irrespective of the vaccine composition as assessed by the cardinal signs of inflammation at the injection site. After the first vaccination, the pain process was similar at 6 h among the three vaccinated heifer groups, but the frequency of animals was higher in the Vaccine B group. Overall, cardinal signs were more intense in the Vaccine B and Vaccine C group than those in Vaccine A and control group. Heat and rubor (redness) are the result of increased blood-flow as a result of increased blood vessel dilatation, which allows the separation of endothelial cells that line the blood vessel, leading to extravasation of fluid, blood proteins and tissue accumulation, edema, all sequalae associated with inflammation. The pain is a result of compression of the local nerve fibers due to the accumulation of fluids and cells [28,29]. These mechanisms may result in the increased pain intensity in heifers that received Vaccine B.

In this study, there was increased localized inflammation in the Vaccine B group at 24 h after first vaccination. Vaccine B and Vaccine C had higher local reactivity than Vaccine A or control groups. Tissue damage has been seen in animals vaccinated with oil-in-water adjuvants and resulting in localized pyogranulomas, infiltrates of intact and necrotic neutrophils, epithelioid macrophages with vacuolated cytoplasm, and more externally, abundant connective tissue between lymphocytes and plasma cells [30].

Higher respiratory signs were seen in heifers vaccinated with vaccines containing oil-in-water (Vaccine B) or Amphigen and QAD (Vaccine C) adjuvants at 48 h and 72 h after the booster. The overproduction of locally produced cytokines may result in a “cytokine storm”. Local inflammation associated with a cytokine storm may result in a systemic response distributed throughout the body and reaching other tissues, such as the lungs and GI tract. The proinflammatory cytokines, IL-6 and IL-1β are found in bronchoalveolar lavages in patients with lung injury [31]. Acute lung injury is a common consequence of this exacerbated cytokine production in humans [32].

The rectal temperature of the vaccinated heifers was higher than the control group at 24 h after the primary vaccination and 24 h after booster vaccination, but the highest rectal temperatures were observed in Vaccine C heifers. The presence of thermosensitive live strain of BHV-1 in vaccine C and the adjuvant formulation probably induced intense local inflammation with the exacerbated release of proinflammatory cytokines IL-1β, IL-6 and TNF-α, circulating reach the hypothalamus and other cerebral branches, stimulating the production of prostaglandin-E2, a substance that induces hyperthermia [7,8,11].

Vaccine C had the higher leukogram response: Increasing platelet, basophil (%), and eosinophil (%) counts, especially following the booster immunization. The general profile of leukocytes types characterized by leukocytosis, neutrophilia, lymphopenia, monocytosis and eosinophilia suggest a possible effect of cortisol, even in the non-vaccinated. The eosinophil count in vaccinated heifers was lower than those unvaccinated between 6 h and 168 h after the first dose, which the present study suggests can be due to the migration of these blood cells to the tissues. Eosinophils promote resolution of inflammation by blocking polymorphonuclear cells infiltration to sites of injury and/or modulating macrophage phenotype through cytokines (IL-4 and IL-13) and/or lipid mediators (protectin D1—PD1 and resolvins—RvE3) [33].

The number of platelets was higher in heifers vaccinated with Vaccine B at 6 h after the first dose of the vaccine and at 72h after the booster shot. This phenomenon has not yet been reported in the current literature following vaccination. It can be hypothesized that proinflammatory cytokines could act in the bone marrow by increasing erythropoiesis as of myeloid cells. Another possibility for increased platelets would be the increase of the splenic contraction mediated by cortisol and release of epinephrine in cases of stress. The spleen sequestrates from a quarter to about two-thirds of total platelets, releasing these cells into the circulatory compartment to increase the number of red blood cells in the bloodstream and increase the supply of oxygen in stressful situations [34].

Iron concentrations in control heifers were higher than those in vaccinated heifers, especially Iron concentrations in control heifers were higher than those in vaccinated heifers, especially compared to the Vaccine C group, between 6 h and 48 h after vaccination. The lower availability of serum iron in vaccinated heifers can be explained by the action of hepcidin to limit the multiplication of iron-dependent extracellular microorganisms [35]. Hepcidin is an antimicrobial peptide mediator of innate immunity synthesized by hepatocytes, which is regulated by IL-6, which induces the transcription of the hepcidin gene through the activation of hepcidin signal transducer and activator of transcription 3 (STAT-3). Intracellular absorbed iron binds to ferritin, targeting the basolateral membrane, where ferroportin is present in enterocytes, macrophages and hepatocytes. Ferroportin are proteins required to transport iron into the plasma [36,37].

It is challenging to distinguish whether the signs of stress observed in this study are due to simple management of cattle and/or pain on vaccination, but the major leukogram changes were observed in vaccinated heifers with Vaccine B after the first dose and Vaccine C after the second dose. The decrease in leukocytes, neutrophils, eosinophils and monocytes values after 24 h post-vaccination also indicates potential migration of these cells to tissues facilitated or not by a possible decrease in cortisol over time.

Measurement of Hp concentration indicates peak of post-vaccinal inflammation, mainly Vaccine C heifers. Hp is one of the most well studied acute phase proteins in cattle. The production is mediated by proinflammatory cytokines derived from innate immune response, can be used as a quantitative indicator of infection, inflammation, tissue injury and stress [38,39]. Cattle immunization with multivalent vaccines against Clostridium also significantly increased serum Hp concentration between 24 h and 48 h post vaccination with a commercial vaccine containing Amphigen/QAD [40]. The same adjuvant composition used in the Vaccine C also had Hp concentrations higher [41]. Vaccinated cows with oil-in-water adjuvanted of foot-and-mouth vaccine had peaked at 24 h after application [42].

The adaptive immune response to BVDV-1 NADL and BVDV-1 Singer of Vaccine A was higher, suggesting that alum mechanism, salt-based adjuvants stimulate the Th2 immune responses, enhanced antigen uptake by antigen presenting cells, NLRP3 inflammasome activation, stimulation and differentiation of CD4^+^ T cells, interact with DC’s membrane and complement activation [43].

The BVDV-2 VS253 SVN revealed only Vaccine C animals responded. Additionally, BHV-1 Abs production was more intense in animals vaccinated with Vaccine C. The adjuvant along with the strain of BHV-1 is a live thermosensitive virus and differ them from Vaccine A and B with inactivated BHV-1 [44]. Thermosensitive vaccines contain a chemically altered live virus capable of replicating only at lower temperatures (30–33 °C) than the body (37 °C), thus the virus cannot replicate in its host, a fact that makes it impossible develop viremias after vaccination with this type of antigen [45].

Other factors in addition to the composition of the vaccines may influence for vaccination response, such as heat stress and food management. This experiment was performed between spring/summer seasons with maximum temperatures of 34 °C, where heat stress could be triggered, which decreases the efficiency of the immune response [46]. In addition, the immune system requires energy, proteins, vitamins and minerals to perform their function. The main source of nutrition for the animals in this research was grass from the pasture (*Brachiaria Brizanthacv.Marandu*). Most Brazilian pastures are deficient and do not provide the ideal levels of micronutrients [47]. Among the most important “microminerals immune” to our research is zinc (Zn), which is directly related to protein synthesis, antibody formation, cell differentiation, skin and mucosal integrity, that is, essential for immune responses innate and humoral [48].

To the best of our knowledge, this is the first study to evaluate the local and systemic effects of different types of adjuvants used in commercial reproductive vaccines on protection against BVDV and BHV-1 in Holstein heifers. The “ideal” adjuvant, which can promote a better specific immune response with weak or no irritation at the injection site, is currently non-existent in Brazil. In this study, there were exacerbated local effects with the oil-in-water adjuvanted commercial vaccine, but the leukogram, as well as the iron and haptoglobin levels indicated that the vaccinated heifers had a systemic inflammatory profile after the vaccinations, stimulated especially by the vaccine formulation containing Amphigen/QAD.

## 5. Conclusions

The findings of this research were based on three different commercial laboratory formulations and also according to the conditions which the study was conducted. In this context, vaccine containing mineral oil or Amphigen/QAD presented greater local reactivity and induced a significant systemic inflammatory response. Vaccinated heifers with Alum and Amphigen/QAD commercial vaccines enhanced humoral immune response against BVDV and BHV-1.

## Figures and Tables

**Table 1:** Vaccine treatment groups and vaccine formulations.

Groups	Adjuvant Composed	Strains	Others Antigens
Vaccine A (*n* = 9)	Aluminum hydroxide (Alhydrogel^®^)	Bovine viral diarrhea virus (BVDV)-1 (Singer) and BVDV-2 inactivated, strains from INTA ^1^ and CEVAN ^2^; bovine herpesvirus 1 (BHV)-1 (Los Angeles) inactivated	*Campylobacter fetus*;* Campylobacter fetus veneralis*; *Leptospira interrogans pomona*; Histophilus somni.
Vaccine B (*n* = 10)	Oil-in-water adjuvant (mineral oil-based)	BVDV-1 and BVDV 2 (inactivated); BHV-1 and BoHV-5 (inactivated)	*Leptospirahardjo*, *L. icterohaemorrhagiae*,* L. Bratislava*,* L. Pomona *and *L. wolfi*
Vaccine C (*n* = 10)	Amphigen and Quil A cholesterol and dimethyl dioctadecylammonium (DDA) bromide (QAD) adjuvant ^®^	BVDV-1 (5960) and BVDV-2 (53,637) inactivated; BHV-1 (RBL106) thermosensitive	*Leptospira canicola*,* L. grippotyphosa*,* L. hardjo*,* L. icterohaemorrhagiae* and *L. pomona*
Control (*n* = 6)	-	Saline Solution	-

^1^ INTA=Instituto Nacional de Tecnología Agropecuaria, Argentina; ^2^ CEVAN = Centro de Virología Animal, Argentina, BVDV = Bovine Viral Diarrhea Virus, BHV-1 = bovine herpesvirus type 1.

## Appendix

**Table A1:** Impact of vaccination against BVDV and BHV-1 with different commercial formulations in animals on a presence of cardinal signs of inflammation.

Variable		First Dose					Second Dose				
		Vaccine A ^2^	Vaccine B ^3^	Vaccine C ^4^	Control ^5^	X^2^	Vaccine A ^2^	Vaccine B ^3^	Vaccine C ^4^	Control ^5^	X^2^
Pain	0 h ^1^	0 (0%)	0 (0%)	0 (0%)	0 (0%)	-	0 (0%)	0 (0%)	0 (0%)	0 (0%)	-
	6 h	7 (78%)	7 (70%)	7 (70%)	0 (0%)	0.012	8 (89%)	6 (60%)	9 (90%)	0 (0%)	0.001
	24 h	5 (56%)	7 (70%)	6 (60%)	0 (0%)	0.044	6 (67%)	5 (50%)	8 (80%)	0 (0%)	0.015
	48 h	5 (56%)	5 (50%)	3 (30%)	0 (0%)	0.123	7 (78%)	7 (70%)	9 (90%)	0 (0%)	0.002
	72 h	5 (56%)	8 (80%)	3 (30%)	0 (0%)	0.013	3 (33%)	4 (40%)	5 (50%)	0 (0%)	0.224
	168 h	4 (44%)	7 (70%)	3 (30%)	0 (0%)	0.041	1 (11%)	5 (50%)	1 (10%)	0 (0%)	0.042
	Cochran’s	0.004	0.000	0.002	-		0.001	0.001	0.000	-	
Heat ^6^	0 h	0 (0%)	0 (0%)	0 (0%)	0 (0%)	-	0 (0%)	0 (0%)	0 (0%)	0 (0%)	-
	6 h	4 (44%)	4 (40%)	8 (80%)	0 (0%)	0.019	4 (44%)	5 (50%)	7 (70%)	0 (0%)	0.057
	24 h	2 (22%)	7 (70%)	9 (90%)	0 (0%)	0.001	6 (67%)	5 (50%)	5 (50%)	0 (0%)	0.079
	48 h	6 (67%)	7 (70%)	5 (50%)	0 (0%)	0.035	2 (22%)	4 (40%)	6 (60%)	0 (0%)	0.079
	72 h	3 (33%)	8 (80%)	6 (60%)	0 (0%)	0.012	2 (22%)	4 (40%)	3 (30%)	0 (0%)	0.348
	168 h	0 (0%)	2 (20%)	2 (20%)	0 (0%)	0.336	1 (11%)	3 (30%)	0 0%)	0 (0%)	0.148
	Cochran’s	0.006	0.001	0.000	-		0.004	0.025	0.000	-	

^1^ h: hours; ^2^ Vaccine A (*n* = 9); ^3^ Vaccine B (*n* = 10); ^4^ Vaccine C (*n* = 10); ^5^ Control (*n* = 6); ^6^ Heat: Considered 35 °C above; X^2^ = Chi-square test. Differences were considered significant when *p* ≤ 0.05.

**Table A2:** Impact of systemic reaction based on respiratory score, rectal temperature, fecal consistency and sum of health score induced by vaccines against BVDV and BHV-1 with different commercial formulations.

Variables		First Dose					Second Dose				
		Vaccine A ^2^	Vaccine B ^3^	Vaccine C ^4^	Control ^5^	X^2^	Vaccine A ^2^	Vaccine B ^3^	Vaccine C ^4^	Control ^5^	X^2^
Cough	0 h ^1^	0 (0%)	0 (0%)	1 (10%)	0 (0%)	-	0 (0%)	0 (0%)	0 (0%)	0 (0%)	-
	6 h	0 (0%)	0 (0%)	1 (10%)	0 (0%)	0.616	1 (11%)	2 (20%)	1 (10%)	0 (0%)	0.677
	24 h	0 (0%)	1 (10%)	2 (20%)	0 (0%)	0.377	1 (11%)	2 (20%)	3 (30%)	0 (0%)	0.441
	48 h	2 (22%)	0 (0%)	2 (20%)	0 (0%)	0.133	1 (11%)	4 (40%)	1 (10%)	1 (17%)	0.307
	72 h	0 (0%)	3 (30%)	1 (10%)	1 (17%)	0.117	1 (11%)	4 (40%)	1 (10%)	0 (0%)	0.199
	168 h	1 (11%)	2 (20%)	1 (10%)	1 (17%)	0.915	1 (11%)	2 (20%)	3 (30%)	0 (0%)	0.774
	Cochran’s	0.180	0.014	0.940	0.416		0.742	0.193	0.139	0.549	
Nasal discharge	0 h	3 33%)	5 (50%)	6 (60%)	1 (17%)	0.331	4 (44%)	5 (50%)	6 (60%)	0 (0%)	0.116
	6 h	4 (44%)	7 (70%)	6 (60%)	1 (17%)	0.124	7 (78%)	5 (50%)	9 (90%)	3 (50%)	0.169
	24 h	5 (55%)	6 (60%)	6 (60%)	2 (33%)	0.722	5 (56%)	8 (80%)	7 (70%)	3 (50%)	0.561
	48 h	5 (55%)	6 (60%)	7 (70%)	2 (33%)	0.551	5 (56%)	9 (90%)	10 (100%)	5 (83%)	0.067
	72 h	3 (33%)	5 (50%)	6 (60%)	1 (17%)	0.331	6 (67%)	10 (100%)	10 (100%)	4 (67%)	0.051
	168 h	6 (66%)	6 (60%)	7 (70%)	3 (50%)	0.865	5 (56%)	7 (70%)	9 (90%)	2 (33%)	0.116
	Cochran’s	0.539	0.920	0.974	0.709		0.335	0.009	0.052	0.044	
Ocular discharge	0 h	0 (0%)	0 (0%)	0 (0%)	0 (0%)	-	0 (0%)	4 (40%)	1 (10%)	1 (17%)	0.117
	6 h	0 (0%)	0 (0%)	0 (0%)	0 (0%)	-	2 (22%)	4 (40%)	2 (20%)	1 (17%)	0.742
	24 h	3 (33%)	2 (20%)	0 (0%)	1 (17%)	0.116	2 (22%)	5 (50%)	5 (50%)	2 (33%)	0.546
	48 h	2 (22%)	2 (20%)	3 (30%)	1 (17%)	0.926	4 (44%)	8 (80%)	8 (80%)	3 (50%)	0.233
	72 h	2 (22%)	0 (0%)	2 (20%)	1 (17%)	0.334	4 (44%)	6 (60%)	6 (60%)	2 (33%)	0.246
	168 h	1 (11%)	6 (60%)	4 (40%)	2 (33%)	0.178	3 (33%)	4 (40%)	3 (30%)	1 (17%)	0.808
	Cochran’s	0.079	0.002	0.018	0.543		0.218	0.155	0.014	0.649	
Rectal temperature	0 h	4 (44%)	1 (10%)	7 (70%)	0 (0%)	0.462	3 (33%)	1 (10%)	3 (30%)	0 (0%)	0.152
	6 h	2 (22%)	5 (50%)	4 (40%)	0 (0%)	0.151	1 (11%)	4 (40%)	6 (60%)	0 (0%)	0.462
	24 h	0 (0%)	0 (0%)	1 (10%)	1 (17%)	0.151	0 (0%)	0 (0%)	4 (40%)	0 (0%)	0.152
	48 h	0 (0%)	0 (0%)	0 (0%)	0 (0%)	-	0 (0%)	0 (0%)	0 (0%)	0 (0%)	-
	72 h	0 (0%)	0 (0%)	0 (0%)	0 (0%)	-	0 (0%)	0 (0%)	0 (0%)	0 (0%)	-
	168 h	1 (11%)	0 (0%)	0 (0%)	0 (0%)	-	0 (0%)	0 (0%)	0 (0%)	0 (0%)	-
	Cochran’s	0.569	0.344	0.278	0.444		0.101	0.189	0.152	-	
Fecal consistency	0 h	0 (0%)	0 (0%)	0 (0%)	0 (0%)	-	0 (0%)	0 (0%)	0 (0%)	0 (0%)	-
	6 h	0 (0%)	0 (0%)	0 (0%)	0 (0%)	-	1 (11%)	0 (0%)	1 (10%)	0 (0%)	0.677
	24 h	0 (0%)	0 (0%)	0 (0%)	0 (0%)	0.462	0 (0%)	1 (10%)	0 (0%)	0 (0%)	0.416
	48 h	3 (33%)	0 (0%)	0 (0%)	2 (33%)	0.190	1 (11%)	0 (0%)	0 (0%)	1 (17%)	0.661
	72 h	2 (22%)	1 (10%)	0 (0%)	1 (17%)	0.211	0 (0%)	0 (0%)	0 (0%)	0 (0%)	-
	168 h	0 (0%)	0 (0%)	0 (0%)	0 (0%)	0.396	2 (22%)	0 (0%)	0 (0%)	0 (0%)	0.876
	Cochran’s	0.093	0.594	-	0.221		0.127	0.156	0.113	0.100	
Sum of health score	0 h	2 (22%)	0 (0%)	1 (10%)	0 (0%)	0.300	1 (11%)	3 (30%)	1 (10%)	0 (0%)	0.356
	6 h	1 (11%)	1 (10%)	2 (20%)	0 (0%)	0.677	1 (11%)	3 (30%)	5 (50%)	0 (0%)	0.990
	24 h	1 (11%)	1 (10%)	3 (30%)	0 (0%)	0.356	1 (11%)	3 (30%)	4 (40%)	0 (0%)	0.218
	48 h	4 (44%)	1 (10%)	2 (20%)	1 (17%)	0.116	1 (11%)	6 (60%)	6 (60%)	2 (33%)	0.024
	72 h	2 (22%)	3 (30%)	1 (10%)	1 (17%)	0.725	3 (33%)	5 (50%)	1 (10%)	0 (0%)	0.081
	168 h	1 (11%)	3 (30%)	4 (40%)	1 (17%)	0.489	2 (22%)	3 (30%)	3 (30%)	0 (0%)	0.501
	Cochran’s	0.472	0.119	0.472	0.629		0.416	0.501	0.044	0.044	

^1^ h: hours; ^2^ Vaccine A (*n* = 9); ^3^ Vaccine B (*n* = 10); ^4^ Vaccine C (*n* = 10); ^5^ Control (*n* = 6); X^2^ = Chi-square test. Differences were considered significant when *p* ≤ 0.05.

**Table A3:** Clinical pathology evaluated by absolute mean values (10^3^/µL) obtained in the counts of leukocytes, platelets and serum iron of animals vaccinated with vaccines against BVDV and BHV-1 with different commercial formulations.

Variables		First Dose				Second Dose			
		Vaccine A ^2^	Vaccine B ^3^	Vaccine C ^4^	Control ^5^	Vaccine A ^2^	Vaccine B ^3^	Vaccine C ^4^	Control ^5^
WBC ^6^(10^3^/µL)	6 h ^1^	16.63	17.22 ^a^	16.07	13.47	15.89 ^A,b,a^	15.80 ^A,B,a^	18.36 ^A,a^	13.50 ^B,a^
	24 h	14.64	13.66 ^b^	16.04	13.60	13.84 ^a^	12.58 ^b^	14.40 ^b^	11.80 ^a,b^
	48 h	14.43	12.19 ^b^	14.13	11.63	13.73 ^a^	12.23 ^b^	14.36 ^b^	11.45 ^a,b^
	72 h	13.92	13.41 ^b^	14.30	12.08	13.51 ^a^	12.21 ^b^	14.11 ^b^	10.60 ^a,b^
	168 h	13.70	13.21 ^b^	14.30	14.05	12.07 ^A,B,b^	12.20 ^A,B,b^	13.45 ^A,b^	9.62 ^B,b^
LYMPH ^7^(10^3^/µL)	6 h	7.76	7.01	6.58	6.98	8.69	6.99	6.73	7.42
	24 h	8.43	6.91	6.33	6.13	8.65	7.01	5.96	6.65
	48 h	8.25	6.59	7.39	6.48	8.40	6.51	7.08	6.71
	72 h	8.09	7.37	7.25	6.82	8.45	6.96	7.22	5.98
	168 h	8.11	7.57	7.67	7.45	7.78	6.71	8.19	5.93
MONO ^8^(10^3^/µL)	6 h	2.11 ^a^	1.80	1.72 ^a,b^	2.06 ^a^	1.40	1.32	1.52	1.58
	24 h	2.06 ^a^	1.81	1.99 ^a^	2.22 ^a^	1.00	1.21	1.14	0.97
	48 h	1.86 ^a,b^	1.44	1.71 ^a,b^	1.43 ^b^	1.31	1.23	1.25	1.09
	72 h	1.85 ^a,b^	1.35	1.59 ^a^	1.23 ^b^	1.35	1.24	1.51	1.05
	168 h	1.31 ^b^	1.38	1.24 ^b^	1.31 ^b^	1.02	1.17	1.33	0.76
BAS ^9^(10^3^/µL)	6 h	0.68 ^A,B^	0.68 ^A,B^	0.41 ^B^	1.19 ^A^	0.73	0.90	0.60	1.11
	24 h	0.86	0.94	0.84	1.36	1.01	1.10	1.28	1.27
	48 h	0.53	0.74	0.98	1.02	1.19	1.25	1.29	0.70
	72 h	0.29	0.80	0.65	0.99	1.00	1.08	1.0	0.89
	168 h	0.46	0.70	0.63	0.69	0.87	0.68	0.89	0.65
NEUTR ^10^(10^3^/µL)	6 h	5.80 ^A,B,a^	7.38 ^A,a^	7.28 ^A,a,b^	3.26 ^B,a,b^	5.17 ^B,C,a^	6.54 ^B,a^	9.10 ^A,a^	3.43 ^C,a,b^
	24 h	3.21 ^B,b^	3.80 ^B,b^	6.92 ^A,b^	3.60 ^B,a,b^	2.92 ^B,b^	3.50 ^B,b^	5.83 ^A,b,c^	2.84 ^B,a,b^
	48 h	3.70 ^A,b^	3.16 ^A,b^	3.87 ^A,c^	2.63 ^A,b^	2.91 ^A,b^	3.20 ^A,b^	4.34 ^A,c^	2.94 ^A,a,b^
	72 h	3.54 ^A,B,b^	3.47 ^A,B,b^	4.68 ^A,c^	2.54 ^B,b^	2.63 ^B,b^	3.16 ^A,B,b^	4.17 ^A,c^	2.62 ^B,b^
	168 h	3.68 ^A,b^	3.39 ^A,b^	4.66 ^A,c^	4.60 ^A,b^	2.49 ^A,B,b^	3.70 ^A,B,b^	3.92 ^A,c^	2.16 ^B,b^
EOS ^11^ (10^3^/µL)	6 h	0.54 ^B,a,b,c^	0.60 ^B^	0.42 ^B,b^	1.08 ^A,a,b^	0.75 ^a,b,c^	0.78	0.96 ^a,b^	0.96 ^a,b^
	24 h	0.72 ^B,a,b,c^	0.78 ^B^	0.74 ^B,a,b^	1.40 ^A,a^	1.04 ^a^	0.98	1.39 ^a^	1.12 ^a,b^
	48 h	0.45 ^b,c^	0.65	1.10 ^a,b^	1.00 ^a,b^	1.08 ^a^	0.99	1.44 ^a^	0.60 ^b^
	72 h	0.29 ^B,c^	0.73 ^A,B^	0.70 ^B,a,b^	1.40 ^A,a^	0.98 ^a,b^	0.99	1.06 ^a,b^	0.78 ^a,b^
	168 h	0.40 ^b,c^	0.59	0.68 ^a,b^	0.57 ^b^	0.78 ^a,b,c^	0.68	0.81 ^a,b^	0.60 ^b^
PLAT ^12^(×10^3^/µL)	6 h	214.33 ^A,B^	333.30 ^A^	254.20 ^A,B^	168.17 ^B^	246.56	221.00	208.80	188.00
	24 h	263.67	251.60	235.20	251.33	225.33	227.30	209.10	155.67
	48 h	205.67	275.60	193.70	259.67	204.89	228.60	191.50	178.33
	72 h	220.00	253.70	285.00	264.67	219.67 ^A,B^	241.00 ^A^	201.60 ^A,B^	157.17 ^B^
	168 h	238.11	266.00	235.00	252.50	207.89	246.00	237.70	163.00
Iron (µM/L)	0 h	28 ^A^	26 ^A,B^	23 ^B^	32	23 ^A,b^	27 ^A,a,b^	25 ^A,a^	29 ^A,a,b^
	6 h	27 ^A,B^	26 ^B^	21 ^B^	35	20 ^A,B,b^	16 ^B,c^	14 ^B,b^	25 ^A,b^
	24 h	29 ^A,B^	23 ^B^	15 ^C^	32	21 ^A,B,b^	19 ^B,b,c^	12 ^C,b^	30 ^A,a,b^
	48 h	30 ^A,B^	26 ^A,B^	23 ^B^	32	33 ^A,b,a^	27 ^B,a,b^	14 ^C,b^	38 ^A,a^
	72 h	28 ^A,B^	24 ^B,C^	21 ^C^	33	27 ^A,B,a,b^	26 ^A,B,b^	24 ^B,a^	33 ^A,a,b^

^1^ h: hours; 1h: hours; ^2^ Vaccine A (*n* = 9); ^3^ Vaccine B (*n* = 10); ^4^ Vaccine C (*n* = 10); ^5^ Control (*n* = 6); ^6^ WBC: Total leukocytes; ^7^ LYMPH: Lymphocytes; ^8^ MONO: Monocytes; ^9^ BAS basophils; ^10^ NEUTR: Neutrophils; ^11^ EOS: Eosinophils; ^12^ PLAT: Platelets; capital letters on the same line show difference between treatments; lowercase letters in the same column show difference between times. Differences were considered significant when *p* ≤ 0.05.

**Table A4:** Clinical pathology evaluated by relative mean values (%) obtained in the leukogram of animals vaccinated with vaccines against BVDV and BHV-1 with different commercial formulations.

Variables		First Dose				Second Dose			
		Vaccine A ^2^	Vaccine B ^3^	Vaccine C ^4^	Control ^5^	Vaccine A ^2^	Vaccine B ^3^	Vaccine C ^4^	Control ^5^
LYMPH ^6^(%)	6 h^1^	46.33 ^a^	41.70 ^a^	42.00 ^a,b^	50.33 ^a,b,c^	51.78 ^A,b,a^	44.70 ^B,C,a^	36.70 ^C,a^	55.33 ^A^
	24 h	56.56 ^A,a,b^	49.30 ^B,a,b^	39.50 ^C,a^	44.17 ^B,C,a^	61.44 ^A,a,b^	56.10 ^A,b,c^	41.20 ^B,a,b^	57.33 ^A^
	48 h	57.22 ^a,b^	54.20 ^b^	52.30 ^c^	56.00 ^b,c^	59.44 ^a,b^	52.90 ^a,b,c^	49.40 ^b,c^	59.00
	72 h	58.56 ^b^	55.90 ^b^	50.80 ^b,c^	57.33 ^b,c^	61.11 ^a,b^	57.20 ^c^	51.70 ^c^	56.67
	168 h	56.00 ^a,b^	56.40 ^b^	53.60 ^c^	55.67 ^a,b^	63.33 ^b^	55.30 ^c^	58.90 ^c^	63.17
MONO ^7^(%)	6 h	12.44 ^A,B,a,b^	10.30 ^A,a^	11.20 ^A,a,b^	15.83 ^C,a,b^	9.22	8.70	8.70	11.33
	24 h	13.89 ^A,B,C,a^	13.80 ^A,B,C,b^	13.00 ^A,B,C,a^	16.67 ^C,b^	7.33	9.90	7.70	8.00
	48 h	12.44 ^a,b^	11.50 ^a,b^	11.80 ^a,b^	12.33 ^a,c,d^	9.56	10.30	8.60	9.67
	72 h	13.22 ^A,a,b^	9.40 ^B,a^	11.00 ^A,B,a,b^	10.17 ^A,B,c,d^	9.78	9.90	10.90	9.67
	168 h	9.67 ^b^	10.90 ^a,b^	9.40 ^b^	9.67 ^d^	8.78	9.80	9.30	8.00
BAS ^8^(%)	6 h	0.56 ^a^	0.50	0.40	0.67	0.45 ^A,a^	0.50 ^A,B,a^	0.30 ^A,B,a^	0.83 ^B,^ª
	24 h	1.11 ^b^	0.90	0.50	0.61	0.67 ^A,a,b^	0.70 ^A,a^	0.60 ^A,a^	1.67 ^B,^ª
	48 h	0.37 ^a^	0.80	0.30	0.83	0.44 ^A,a^	1.80 ^B,b^	1.50 ^B,b^	1.00 ^A,B,a^
	72 h	0.67 ^a^	0.90	0.70	0.67	1.33 ^b^	0.70 ^a^	1.00 ^a,b^	1.50ª
	168 h	0.67 ^a^	0.60	0.30	0.96	0.44 ^A,a^	1.10 ^A,B,a,b^	0.70 ^A,B,a^	1.50 ^B,a^
NEUTR ^9^(%)	6 h	36.80 ^A,a^	42.81 ^A,a^	43.40 ^A,a^	25.00 ^B^	35.00 ^B,a,b^	41.36 ^A,B,a^	49.50 ^A,a^	25.33 ^C^
	24 h	23.80 ^B,c,d,e^	30.00 ^B,b^	42.70 ^A,a^	27.00 ^B^	21.70 ^B,c,d,e^	27.81 ^B,b^	41.30 ^A,a^	23.67 ^B^
	48 h	27.00 ^a,b,c,d^	26.00 ^b^	26.90 ^b^	22.67	22.40 ^c,d,e^	27.18 ^b^	30.90 ^b^	25.00 ^A^
	72 h	25.70 ^A,B,b,c^	26.00 ^A,B,b^	31.90 ^A,b^	21.17 ^B^	20.10 ^e^	25.54 ^b^	29.40 ^b^	24.33
	168 h	30.20 ^a,b,c^	26.18 ^b^	31.60 ^b^	29.16	21.30 ^B,d,e^	29.00 ^A,b^	25.20 ^A,B,b^	21.66 ^B^
EOS ^10^(%)	6 h	3.30 ^B,b,c^	3.54 ^B,c^	2.90 ^B,b^	8.78 ^A,a,b,c^	5.10 ^B,a,b,c^	4.81 ^B,b,c^	5.00 ^B,a,b^	7.16 ^A,a,b,c^
	24 h	5.00 ^B,a,b,c^	5.45 ^B,a,b,c^	4.70 ^B,a,b^	9.96 ^A,a,b^	8.20 ^a,b^	7.64 ^a,b^	9.10 ^a^	9.33 ^a,b,c^
	48 h	3.30 ^b,c^	5.82 ^a,b,c^	8.20 ^a^	8.16 ^a,b,c^	8.70 ^A,a^	8.36 ^A,a^	9.40 ^A,a^	5.33 ^B,A,c^
	72 h	2.20 ^B,c^	5.55 ^B,a,b,c^	5.50 ^B,a,b^	10.12 ^A,a^	8.30 ^a^	8.09 ^a,b^	7.10 ^a,b^	7.83 ^a,b,c^
	168 h	3.10 ^c^	5.09 ^a,b,c^	5.50 ^a,b^	5.30 ^A,c^	7.00 ^A,a,b,c^	5.91 ^B,a,b,c^	5.80 ^B,a,b^	5.67 ^B,b,c^

^1^ h: hours; ^2^ Vaccine A (*n* = 9); ^3^ Vaccine B (*n* = 10); ^4^ Vaccine C (*n* = 10); ^5^ Control (*n* = 6); ^6^ LYMPH: Lymphocytes; ^7^ MONO: Monocytes; ^8^ BAS basophils; ^9^ NEUTR: Neutrophils; ^10^ EOS: Eosinophils; capital letters on the same line show difference between treatments; lowercase letters in the same column show difference between times. Differences were considered significant when *p* ≤ 0.05.
